# DNAJB6b-enriched small extracellular vesicles decrease polyglutamine aggregation in *in vitro* and *in vivo* models of Huntington disease

**DOI:** 10.1016/j.isci.2021.103282

**Published:** 2021-10-14

**Authors:** Bhagyashree S. Joshi, Sameh A. Youssef, Reinier Bron, Alain de Bruin, Harm H. Kampinga, Inge S. Zuhorn

**Affiliations:** 1 Department of Biomedical Engineering, University of Groningen, University Medical Center Groningen, A. Deusinglaan 1, 9713 AV Groningen, the Netherlands; 2 Department of Biomolecular Health Sciences, Faculty of Veterinary Medicine, Utrecht University, Utrecht, the Netherlands; 3 Department of Molecular Genetics, Department of Pediatrics, University Medical Center Groningen, Groningen, the Netherlands; 4 Department of Biomedical Sciences of Cells and Systems, University of Groningen, University Medical Center Groningen, Groningen, the Netherlands

**Keywords:** Cell biology, Molecular neuroscience, Molecular physiology

## Abstract

Huntington disease (HD) is a devastating neurodegenerative disorder characterized by aggregation of huntingtin (HTT) protein containing expanded polyglutamine (polyQ) tracts. DNAJB6, a member of the DNAJ chaperone family, was reported to efficiently inhibit polyQ aggregation *in vitro*, in cell models, and *in vivo* in flies, xenopus, and mice. For the delivery of exogenous DNAJB6 to the brain, the DNAJB6 needs to be protected against (enzymatic) degradation and show good penetration into brain tissue. Here, we tested the potential of small extracellular vesicles (sEVs) derived from neural stem cells (NSCs) for delivery of DNAJB6 as anti-amyloidogenic cargo. Administration of sEVs isolated from DNAJB6-overexpressing cells to cells expressing expanded polyQ tracts suppressed HTT aggregation. Furthermore, intrathecal injection of DNAJB6-enriched sEVs into R6/2 transgenic HD mice significantly reduced mutant HTT aggregation in the brain. Taken together, our data suggest that sEV-mediated molecular chaperone delivery may hold potential to delay disease onset in HD.

## Highlights

• Passive and active loading of neural-stem-cell-derived sEVs with DNAJB6 chaperone
• DNAJB6-sEVs suppressed polyQ aggregation in an *in vitro* model of Huntington disease
• GFP-DNAJB6-sEVs reduced Huntingtin (HTT) aggregation in the striatum of R6/2 HD mice

## Graphical abstract

**Figure:**
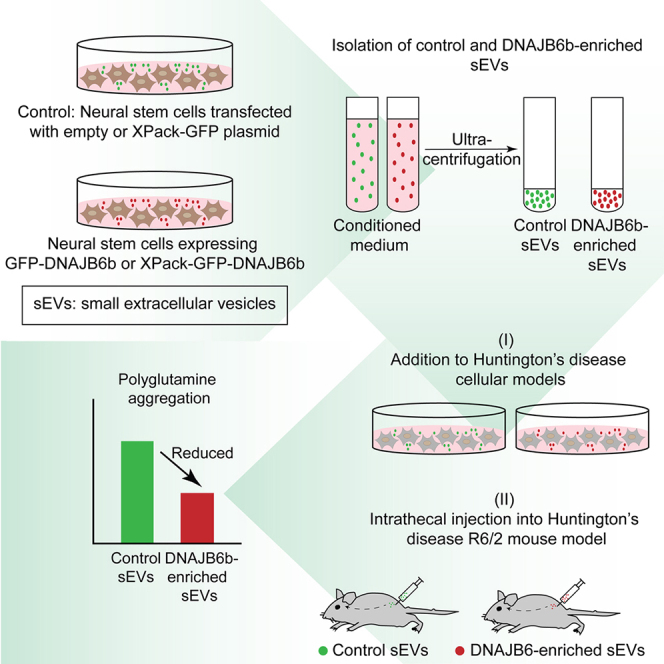

## Introduction

Generation and maintenance of correct protein folding is not only essential for proper functioning of proteins but also key to prevent accumulation of protein aggregates that may gain toxic functions, eventually resulting in cellular dysfunction or cell death (Balchin et al., 2016; Jahn and Radford, 2005). Many neurodegenerative diseases, including Huntington disease (HD), are caused as a result of aggregation-associated proteotoxicity and associated neuronal death (Balch et al., 2008; Balchin et al., 2016). HD is a monogenic disease caused by a genetic mutation in the huntingtin (HTT) gene, which results in an abnormal expansion (>35) of a polyQ encoding CAG repeat (O'Donovan, 1993). The expanded polyQ-containing Htt protein has an increased aggregation propensity, which is proportional to the length of the repeat and inversely correlated to the age of onset of the progressive degeneration of neurons in specific brain areas (Takeuchi and Nagai, 2017).

As being key regulators of protein homeostasis, molecular chaperones hold promise as a therapeutic strategy in HD and other protein instability diseases (Labbadia et al., 2012). Indeed, several molecular chaperones, especially those belonging to the co-chaperone family of DNAJ proteins, e.g. DNAJB2 (Howarth et al., 2007) and DNAJB6 (Chai et al., 1999; Kakkar et al., 2016; Labbadia et al., 2012; Takeuchi et al., 2015; Warrick et al., 1999), were found not only to be effective in reducing polyQ aggregation but also to delay the onset of disease in transgenic mouse models. DNAJB6 is among the most powerful modifiers of polyQ aggregation *in vitro* and *in vivo* (Bason et al., 2019).

For therapeutic translation of DNAJB6 as target in HD therapy, a number of options are available, including e.g. gene therapy. However, this poses challenges for its long-term use owing to safety issues associated with nucleic acid therapeutics such as off-target effects and little control over protein expression levels (Ross and Tabrizi, 2011; Takeuchi and Nagai, 2017). Such challenges may potentially be overcome by direct delivery of DNAJB6 as protein. However, the scope of direct protein administration therapy is limited because the injected naked recombinant protein is vulnerable to degradation and needs to find its way to the cell type and cellular compartment where the functional effect is required (Balch et al., 2008). Therefore, an encapsulating agent that can protect the protein as well as confer it the specificity of uptake without compromising its activity is required for efficient therapeutic protein delivery.

Extracellular vesicles (EVs) have shown great promise as natural drug delivery vehicles for various biomolecules, including proteins (Sterzenbach et al., 2017; Zomer et al., 2015). These endogenously produced nanovesicles act as mediators of intercellular communication by biomolecular signals such as lipids, proteins, and nucleic acids (Schneider and Simons, 2013). Owing to their capability of long-range communication, low immunogenicity, and target cell specificity (Ha et al., 2016; Hoshino et al., 2015; Mathieu et al., 2019; Thery et al., 2002; Tkach and Thery, 2016; Wiklander et al., 2015), EVs may improve both brain distribution of their cargo as well as neuronal uptake.

Here, we address the potential of neural-stem-cell (NSC)-derived sEV-mediated delivery of the molecular chaperone DNAJB6 in cellular and mouse HD models for suppression of polyQ aggregation. By tagging DNAJB6 with a commercially available XPack tag (XP), we loaded DNAJB6 into EVs. Exogenously applied DNAJB6-loaded sEVs suppressed polyQ aggregation in cellular models. Furthermore, when injected intrathecally in R6/2 transgenic HD mice, DNAJB6-loaded sEVs led to a significant decrease in polyQ aggregation in striatum, the most affected brain area in HD (Ross and Tabrizi, 2011). Further exploration is needed to evaluate the therapeutic value of sEV-mediated DNAJB6 delivery to the brain to treat HD.

## Results

### sEVs passively loaded with DNAJB6 suppress polyQ aggregation in mRFP-Htt(Q74) HEK293T cells

**Figure 1:**
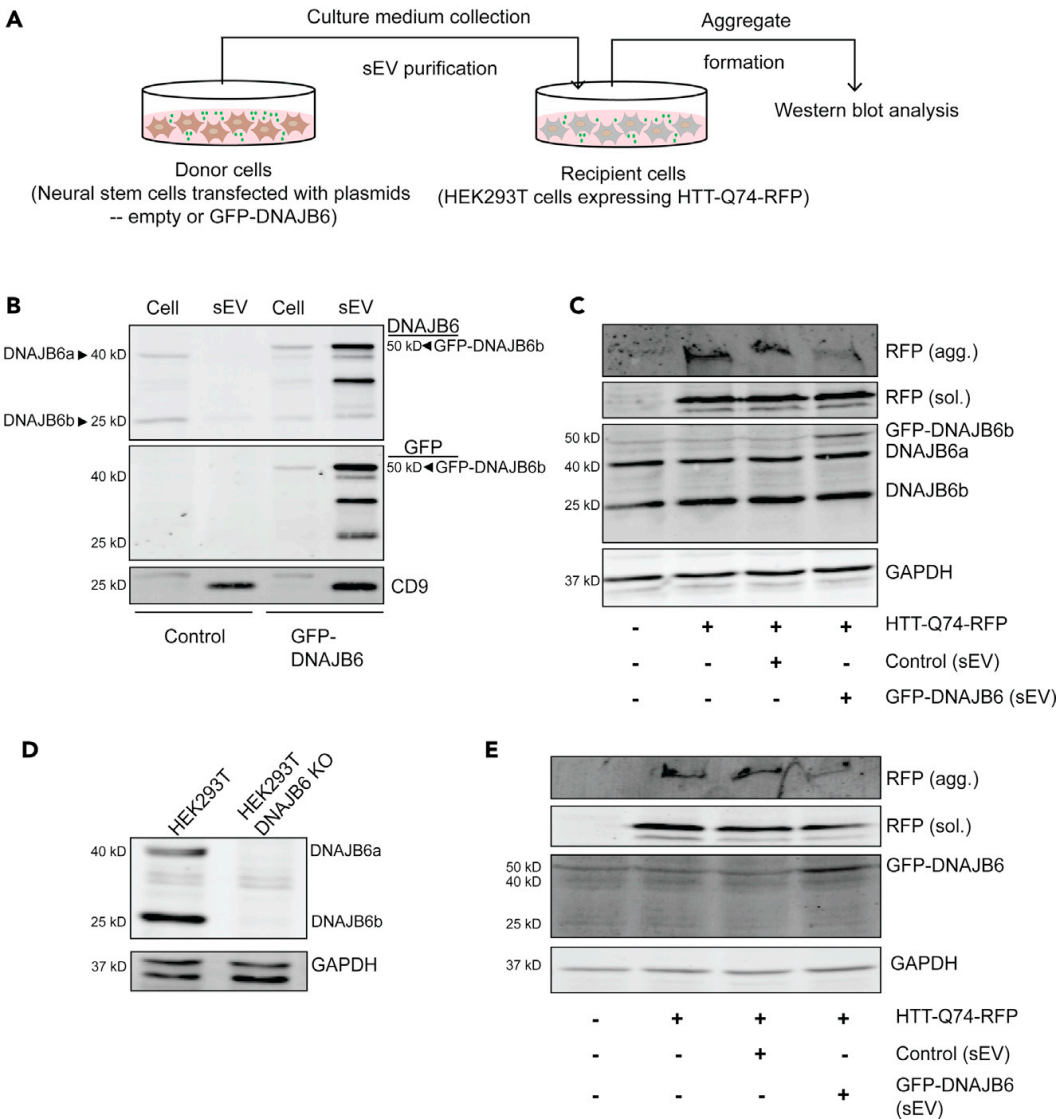
SEVs passively loaded with DNAJB6 suppress polyQ aggregation *in vitro* (A) Experimental strategy for assessing the effect of passive loading of DNAJB6 in sEVs on HTT protein levels in HTT-Q74-RFP HEK293T cells. SEVs isolated from NSCs transfected with empty or GFP-DNAJB6 vector are administered to recipient cells expressing HTT-Q74-RFP. After 48 h, the extent of insoluble polyQ aggregation is assessed using western blotting. (B) Western blot analysis of donor cells and their derived sEVs (20 μg protein per sample per lane). SEVs derived from empty vector-transfected cells show no enrichment of DNAJB6 with respect to donor cells, whereas DNAJB6 in significantly enriched in sEVs derived from GFP-DNAJB6 transfected cells when compared with donor cells. As expected, GFP signal is present only in GFP-DNAJB6 transfected cells and sEVs and is congruent with the DNAJB6 signal. SEV marker CD9 is highly enriched in sEVs compared with donor cells. (C) Western blot analysis of cells expressing HTT-Q74-RFP treated with control or GFP-DNAJB6 sEVs or left untreated. RFP (agg) indicates insoluble HTT-Q74-RFP aggregates, whereas mRFP (sol) shows the soluble form of HTT-Q74-RFP. Note the presence of GFP-DNAJB6b (50 kDa) in cells (in addition to DNAJB6a and DNAJB6b as designated) incubated with sEVs derived from GFP-DNAJB6b-transfected cells together with suppression of HTT-Q74-RFP aggregation. GAPDH is used as loading control (30 μg protein per sample per lane). (D) Western blot analysis of HEK293T WT and DNAJB6 KO cells for the presence (WT) and absence (KO) of DNAJB6. (30 μg protein per sample per lane). (E) Western blot analysis of HEK293T cells expressing HTT-Q74-RFP treated with control or GFP-DNAJB6 sEVs or left untreated. RFP (agg) shows insoluble HTT-Q74-RFP aggregates, whereas RFP (sol) shows soluble HTT-Q74-RFP, blotted with anti-RFP antibody. Note the presence of GFP-DNAJB6 in cells incubated with sEVs derived from GFP-DNAJB6-transfected cells and corresponding suppression of HTT-Q74-RFP aggregation. GAPDH is used as loading control for both the blots (20 μg protein per sample per lane). *Cell*: whole-cell lysate; *sEV*: sEV lysate.

To evaluate the potential of NSC-derived sEVs for delivery of the molecular chaperone DNAJB6 into cells, we first prepared DNAJB6-loaded sEVs through the overexpression of DNAJB6 in NSCs. We specifically overexpressed the isoform b of DNAJB6 because a differential role for the two isoforms of DNAJB6 has been suggested by experiments, demonstrating that DNAJB6a (the longer, nuclear localized isoform, 40kDa) effectively suppresses nuclear poly-Q protein aggregation but is ineffective with cytoplasmic aggregation, whereas DNAJB6b (the shorter, cytoplasmic isoform, 25kDa) is a potent suppressor of cytoplasmic as well as nuclear poly-Q aggregation (Hageman et al., 2010; Meng et al., 2016). For this purpose, C17.2 cells were transfected with empty vector (pcDNA5/FRT/TO) and GFP-DNAJB6-encoding plasmid (Figure 1A). Diffuse nuclear and cytosolic GFP staining in GFP-DNAJB6 overexpression cells was consistent with the endogenous localization of DNAJB6 (Figure S1). sEVs derived from GFP-DNAJB6-overexpressing cells contained high amounts of DNAJB6 in comparison to their respective donor cells, whereas sEVs collected from empty-vector-transfected cells contained low levels of DNAJB6, as was revealed by western blot analysis (Figure 1B). Three main DNAJB6 protein bands were detected in lysates of sEVs derived from GFP-DNAJB6-transfected cells, with molecular weights of approximately 30kDa, 40kDa, and 50kDa (Figure 1B). Because GFP has a molecular weight of 25kDa, the 50kDa band corresponds to GFP-DNAJB6 (isoform b). We speculate that the 30 and 40 kDa bands represent degradation products. Taken together, the data indicate that the b isoform (25 kDa) of DNAJB6 was predominantly present in sEVs, whereas donor cells contained both the a (40 kDa) and b (25 kDa) isoforms. This is in line with the differential location of the DNAJB6 isoforms (Hageman et al., 2010).

To test whether DNAJB6-loaded sEVs may impede on the formation of polyQ aggregates in recipient cells, HEK293T cells were transfected with pmRFP-Q74, and formation of high-molecular-weight aggregates was assessed in the absence and presence of control and GFP-DNAJB6 sEVs. High-molecular-weight aggregates were present in untreated mRFP-Q74 HEK293T cells, as revealed by the presence of RFP in the stacking gel upon SDS-PAGE of the lysate of mRFP-Q74 HEK293T cells (Figure 1C). Incubation of mRFP-Q74 HEK293T cells with sEVs collected from empty-vector-transfected cells had no effect on the amount of polyQ aggregates in the stacking gel, whereas sEVs collected from GFP-DNAJB6-transfected cells significantly suppressed polyQ aggregation, without having an effect on the soluble polyQ expression levels (Figure 1C). Of note, mRFP-Q74 HEK293T cells treated with GFP-DNAJB6 EVs showed an additional DNAJB6 protein band at a molecular weight of 50 kDa, representing GFP-DNAJB6 (Figure 1C). Overall, these results indicate that sEVs passively loaded with GFP-DNAJB6 transfer GFP-DNAJB6 into recipient mRFP-Q74 HEK293T cells and reduce polyQ aggregation. To validate if the anti-aggregation effect in Htt(Q74)-expressing cells is not caused by factors in the EVs that stimulate endogenous DNAJB6, sEVs were administered to DNAJB6 knock-out HEK293T cells expressing mRFP-Q74. First, we confirmed that the DNAJB6 KO cells were devoid of both the DNAJB6 isoforms (Figure 1D). Next, DNAJB6 KO HEK293T cells were transfected with pmRFP-Q74, and formation of high-molecular-weight aggregates was assessed in the absence and presence of control and GFP-DNAJB6 sEVs. After addition of sEVs, the amount of polyQ aggregates was reduced only when sEVs derived from GFP-DNAJB6 cells were added, further strengthening the premise of DNAJB6 transfer through sEVs being responsible for the polyQ aggregate reducing effect (Figure 1E). However, we cannot fully exclude that sEVs derived from GFP-DNAJB6-overexpressing cells, in addition to GFP-DNAJB6, contain other components that may affect polyQ aggregation.

Quantification of the amount of DNAJB6 in control and GFP-DNAJB6 sEVs on the western blot in Figure 1B revealed that GFP-DNAJB6 sEVs contained approximately five times the amount of DNAJB6b as compared with control sEVs (Table S1). In an attempt to further improve the reducing effect of GFP-DNAJB6 sEVs on mRFP-Q74 aggregation, we set out to enhance the loading of DNAJB6 into sEVs.

### Generation of DNAJB6-enriched sEVs from C17.2 neural stem cells through active loading

**Figure 2:**
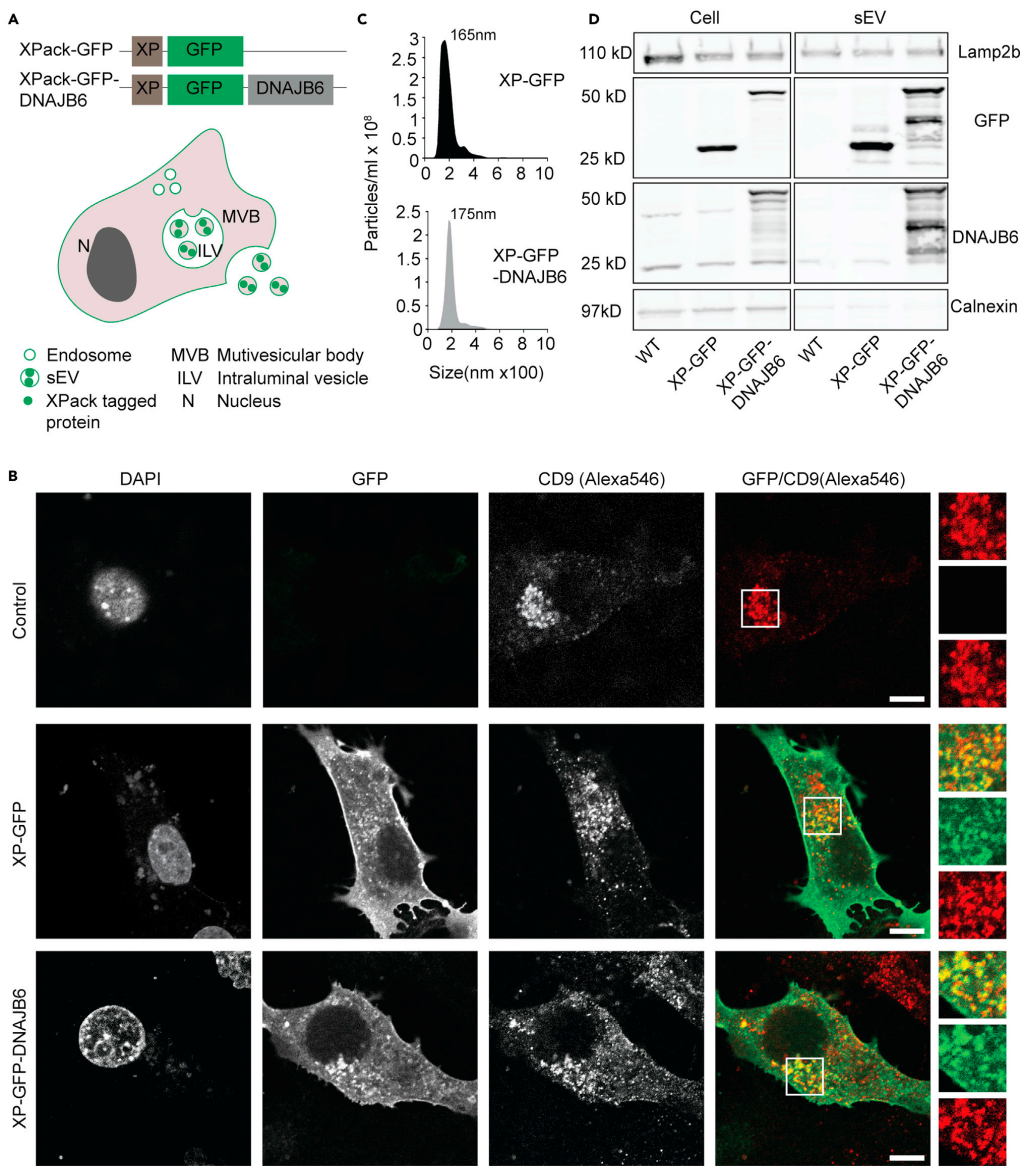
Generation and characterization of XP-GFP and XP-GFP-DNAJB6 sEVs (A) XP-GFP and XP-GFP-DNAJB6 constructs used in this study. XP-tagged proteins are directed into sEVs. (B) Representative images of donor cells expressing XP-GFP or XP-GFP-DNAJB6. Wild-type cells serve as control. XP-tagged proteins colocalize with CD9, an MVB marker protein. The insets show enlarged view of boxed areas. DAPI was used for nuclear staining. (C) Size distribution of sEVs measured by NTA. (D) Western blot analysis of whole-cell and purified sEV lysates (20 μg protein per sample per lane). XP-tagged GFP and GFP-DNAJB6 proteins are enriched in sEV fractions as compared with respective donor cells. WT sEVs isolated from untransfected cells serve as a control. Endogenous DNAJB6b is present in cell lysates and at low amounts in sEV fractions. Note the high amount of DNAJB6 present in XP-GFP-DNAJB6 sEV fraction. SEV marker Lamp2b is present in all sEV samples at similar levels. The endoplasmic reticulum marker protein calnexin is nearly absent in all sEV fractions. *XP*: XPack; *sEV*: purified sEV lysate; *Cell*: whole-cell lysate. Scale bar: 10 μm.

In order to enhance the loading of DNAJB6 into sEVs, we used a commercial XPack protein packaging vector (System Biosciences, Mountain View, CA, USA). With this vector proteins can be targeted to sEVs and microvesicles by altering their affinity for the plasma membrane and/or degree of oligomerization (Figure 2A) (Shen et al., 2011; Yang and Gould, 2013). sEV donor cell lines were created through the stable expression of XP-GFP and XP-GFP-DNAJB6 constructs in C17.2 cells, for the generation of control and DNAJB6-enriched sEVs, respectively (Figure 2A). GFP was fused at the N-terminus of DNAJB6, as the DNAJB6 C-terminus is important for its chaperone activity (Hageman et al., 2010; Kakkar et al., 2016).

The GFP signal in XP-GFP- and XP-GFP-DNAJB6-expressing cells showed plasma membrane staining and a punctate staining pattern in the cytosol, most likely reflecting endosomal structures (Figure 2B). Many of the GFP-positive structures colocalized with CD9, a marker used to identify MVBs (Figure 2B) (Bobrie et al., 2012). This suggests that the XP peptide tag targeted the recombinant proteins to MVBs, i.e., the site of sEV biogenesis, leading to enrichment of these proteins in sEVs, which was evaluated next. sEVs isolated from the XP-GFP and XP-GFP-DNAJB6 donor cell lines showed similar size distributions as determined by Nanoparticle Tracking Analysis (NTA) (Figure 2C). Western blot analysis revealed significant enrichment of XP-tagged proteins in sEV fractions in comparison to the respective parent donor cells. XP-GFP-DNAJB6 sEVs were enriched in DNAJB6 compared with the donor cells, whereas WT and XP-GFP sEVs did not show enrichment in DNAJB6 (Figure 2D). Of note, endogenous DNAJB6b was detected in all sEV types, including WT sEVs, indicating that endogenous DNAJB6b protein is also inherently loaded into sEVs of C17.2 cells, albeit at low levels. XP tagging of DNAJB6 increased the loading of total DNAJB6b into sEVs by 12.8 and 11.7 times as compared with WT and XP-GFP sEVs, respectively, as determined from the western blot shown in Figure 2D (Table S1). Altogether, these results indicated that sEVs derived from the XP-GFP and XP-GFP-DNAJB6 donor cell lines were similar in size and enriched in XP-tagged proteins and thus suitable to be potentially used as protein delivery systems. Moreover, the active loading of sEVs with proteins by means of the XPack protein packaging vector enhanced the loading of DNAJB6 into sEVs compared with the passive loading via the overexpression of proteins in the cell cytosol (Table S1).

### DNAJB6-enriched sEVs are efficiently internalized by EGFP-Htt(Q74) HEK293T cells

**Figure 3:**
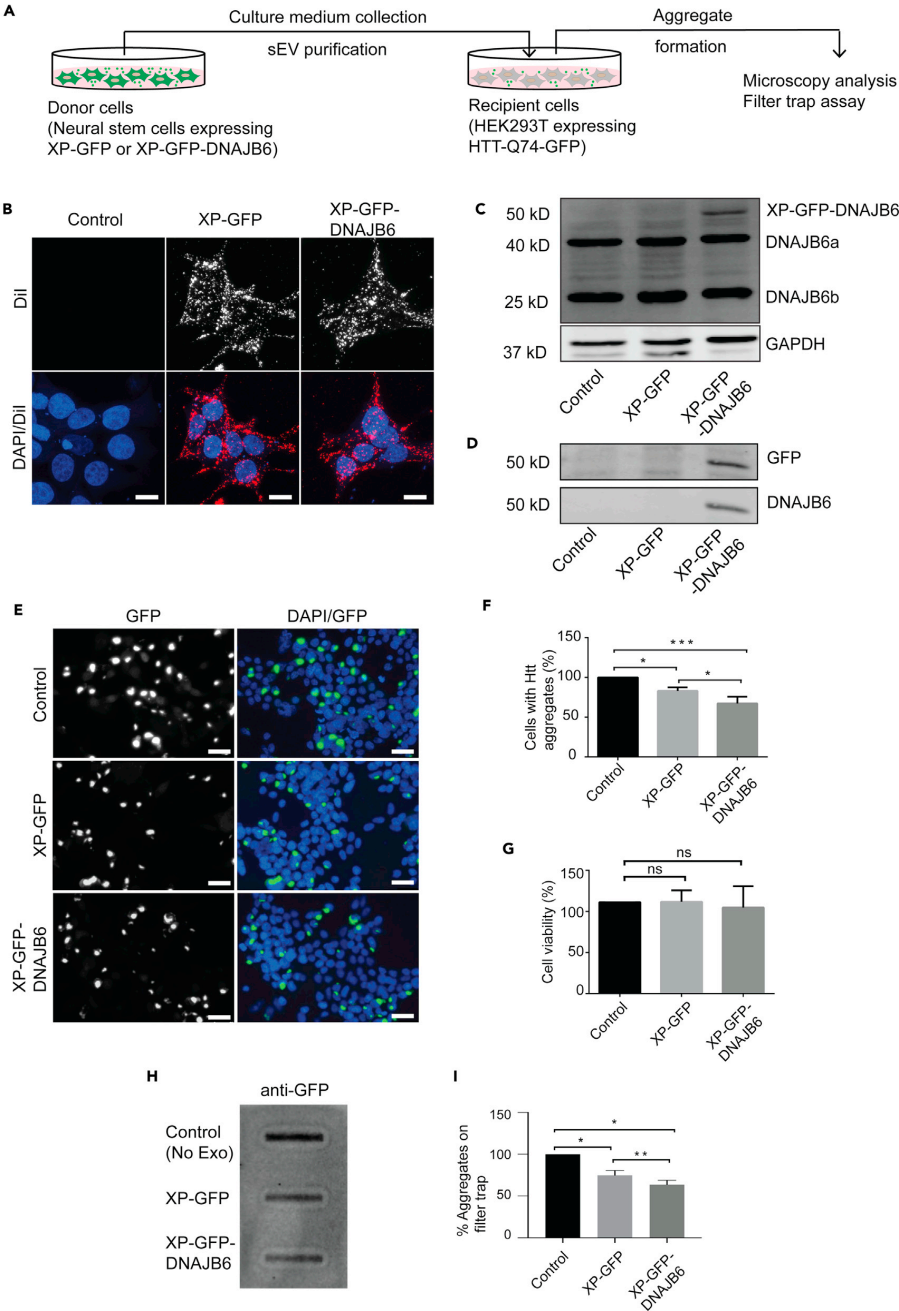
DNAJB6-enriched sEVs suppress polyQ aggregation *in vitro* (A) Schematic representation of experimental strategy for assessing the effect of DNAJB6-enriched sEVs on HTT protein levels in HTT-Q74-GFP HEK293T cells. Purification of sEVs from NSCs expressing XP-GFP or XP-GFP-DNAJB6 followed by their administration to recipient cells expressing HTT-Q74-GFP is depicted. The recipient cells are assessed for aggregate formation with microscopy and filter trap assay 48 h post-sEV addition. (B) Control cells and cells treated for 48 h with XP-GFP or XP-GFP-DNAJB6 sEVs (20 μg/mL in 200μL of medium) labeled with a lipophilic dye DiI. Note sEVs taken up by recipient cells can be seen as red dots. (C) Immunoblotting analysis of DNAJB6 in recipient cells left untreated, treated with XP-GFP or XP-GFP-DNAJB6 sEV fractions. GAPDH was used as a loading control. Note the presence of the exogenously delivered DNAJB6 in XP-GFP-DNAJB6 sEV-treated cells (30 μg protein per sample per lane, n = 2). (D) Immunoblotting analysis of GFP and DNAJB6 in recipient cells left untreated, treated with XP-GFP or XP-GFP-DNAJB6 sEV fractions. Note the presence of GFP and DNAJB6 in XP-GFP-DNAJB6-treated cell lysates at 50kDa, confirming the presence of GFP-DNAJB6 fusion protein (30 μg protein per sample per lane, n = 1). (E and F) Confocal microscopy images and graphical representation of quantification of polyQ aggregation in HTT-Q74-GFP-expressing cells 48 h after the addition of XP-GFP or XP-GFP-DNAJB6 sEVs (50 μg/mL in 1mL of medium). Untreated cells serve as a control (set at 100%) (n = 3). (G) Cell viability of cells expressing HTT-Q74-GFP untreated (control), treated with XP-GFP or XP-GFP-DNAJB6 sEVs (n = 3). (H) Filter trap assay of extracts of cells expressing HTT-Q74-GFP left untreated (control), treated with XP-GFP or XP-GFP-DNAJB6 sEVs (n = 3). (I) Graphical representation of the quantification of insoluble aggregates based on three independent filter trap assays. Data are represented as mean ± SD of three independent experiments (∗p < 0.05, ∗∗p < 0.01; ns, nonsignificant; ANOVA Tukey's post hoc test). DAPI was used for nuclear staining. *XP*: XPack; *sEV*: sEVs. Scale bars: (B) 10 μm and (E) 25 μm.

Next, we set out to examine whether the XP-GFP-DNAJB6 sEVs could suppress polyQ aggregation in cells *in vitro*. To this end, the sEVs were administered to HEK293T cells expressing GFP-tagged Huntingtin protein containing 74 glutamine repeating units (EGFP-Htt(Q74)) (Figure 3A). First, we determined whether XP-GFP and XP-GFP-DNAJB6 sEVs were taken up by EGFP-Q74 cells. For this purpose, sEVs were labeled with a lipophilic dye (DiI) and incubated with EGFP-Q74 cells for 48 h. Both sEV types showed efficient internalization by EGFP-Q74 cells, revealing a punctate fluorescence pattern (Figure 3B) indicative of the involvement of endocytosis in the cellular uptake of the sEVs. The mean fluorescence intensity per cell was similar in cells treated with XP-GFP and XP-GFP-DNAJB6 sEVs, showing that the two sEV types were internalized to a similar extent (Figure S2A). Furthermore, immunoblotting analysis of recipient cells confirmed the presence of the recombinant DNAJB6 protein (50 kDa) together with the two endogenous DNAJB6 isoforms (40 kDa and 25 kDa) in cells incubated with XP-GFP-DNAJB6 sEVs, confirming successful internalization of sEVs (Figure 3C). Moreover, the 50 kDa protein band was positive for both GFP and DNAJB6, confirming the presence of the GFP-DNAJB6 fusion protein in cells incubated with XP-GFP-DNAJB6 sEVs (Figure 3D). As expected, untreated cells and cells treated with XP-GFP sEVs contained endogenous DNAJB6 only, whereas endogenous DNAJB6 levels were comparable in untreated cells and cells treated with either type of sEV (Figure 3C).

### DNAJB6-enriched sEVs abrogate polyQ aggregation in EGFP-Htt(Q74) HEK293T cells

Next, we investigated the effect of DNAJB6-enriched sEVs on protein aggregate formation in recipient cells expressing expanded polyQ (EGFP-Htt(Q74)). Because DNAJB6 is capable of preventing the intracellular aggregation of polyQ peptides, but does not support disaggregation (Hageman et al., 2010), HEK293T cells were incubated with sEVs immediately after their transfection with pDNA encoding EGFP-Htt(Q74) for 4 h. In this way, we assured that sEVs and their cargo were present within the cells before/during EGFP-Q74 expression and subsequent protein aggregate formation. Incubation with XP-GFP-sEVs already significantly suppressed polyQ aggregation in EGFP-Q74 HEK293T cells, showing a 17 ± 4% decrease in the number of EGFP-Q74 foci as compared with untreated cells (Figures 3E and 3F). This is consistent with earlier findings that sEVs from nontransfected cells contain endogenously expressed chaperones, including several members of the DNAJ protein family (DNAJB1, DNAJB2, DNAJB6) (Takeuchi et al., 2015) that all have been shown to have anti-polyQ aggregation activity in cells (Hageman et al., 2010; Howarth et al., 2007; Labbadia et al., 2012). However, recipient cells treated with sEVs containing XP-GFP-DNAJB6, i.e., enriched in DNAJB6, suppressed polyQ aggregation with 33 ± 7% when compared with untreated cells (Figure 3F). To corroborate these results, we used the filter trap assay (Wanker et al., 1999) for biochemical detection of polyQ aggregates. These data confirmed the reduction in insoluble aggregates when cells were incubated with XP-GFP sEVs (by 25 ± 6%) as well as the enhanced reduction when using XP-GFP-DNAJB6 sEVs (by 36 ± 5%) (Figures 3H, 3I, and S2B).

### DNAJB6-enriched sEVs abrogate polyQ aggregation in R6/2 mice

**Figure 4:**
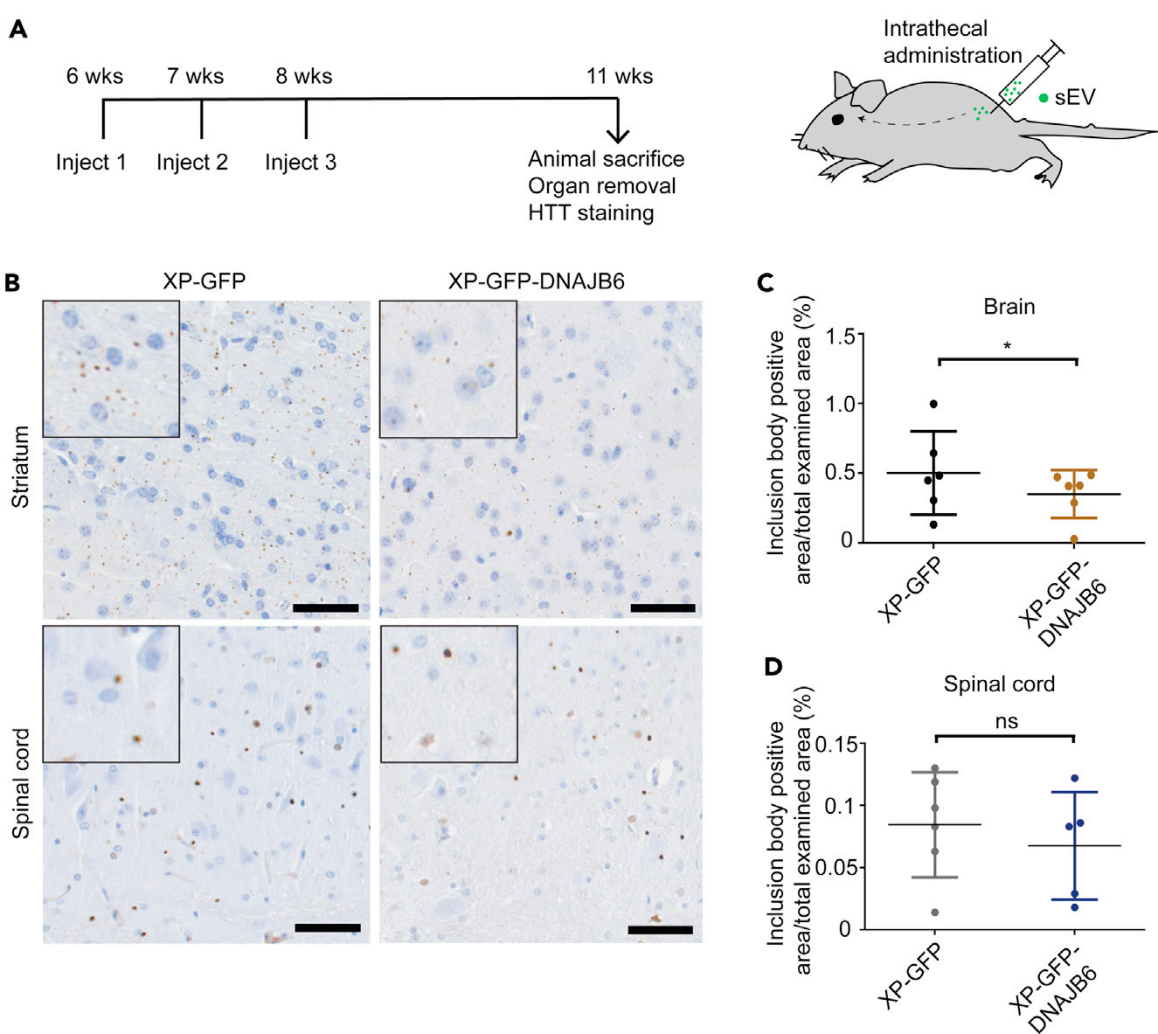
DNAJB6-enriched sEVs suppress polyQ aggregation *in vivo* (A) Experimental workflow for sEV administration to R6/2 mice and HTT staining of brain and spinal cord tissue. 10 μL of a 5mg/mL sEV (XP-GFP-DNAJB6 or XP-GFP) formulation (total 50 μg protein) was used per injection. (B) Immunohistochemistry images of HTT aggregates in striatum and spinal cord tissues. Note the decrease in aggregates (brown spots) in tissues of XP-GFP-DNAJB6 sEV-treated animals. Insets in (B) show magnified view of selected cells. (C and D) Quantification of HTT aggregates in brain and spinal cord tissues for XP-GFP and XP-GFP-DNAJB6 sEV-treated animals. HTT aggregation is significantly reduced in striatum in XP-GFP-DNAJB6 sEV-treated animals as compared with animals with XP-GFP sEV treatment. Data are represented as mean ± SD of n = 6 animals (∗p < 0.05; ns, nonsignificant; Wilcoxon test). Hematoxylin was used for nuclear staining. *XP*: XPack. Scale bar: 50 μm.

To test the *in vivo* delivery potency of DNAJB6-enriched sEVs, we examined their use in reducing polyQ aggregation in R6/2 HD transgenic mice. The R6/2 mouse model is a widely used HD model that recapitulates molecular features of HD over an accelerated time course (Davies et al., 1997; Mangiarini et al., 1996). For *in vivo* experiments, XP-GFP sEVs were used as control, in order to investigate the effect of overexpressed DNAJB6 against the background of endogenous EV content. At 6 weeks of age, R6/2 mice have not yet formed inclusion bodies (Davies et al., 1997). Because DNAJB6 is capable of preventing the intracellular aggregation of polyQ peptides, but does not support disaggregation (Hageman et al., 2010), we administered XP-GFP- or XP-GFP-DNAJB6 sEVs to R6/2 mice via an intrathecal route starting at 6 weeks of age. Two more injections were given at 7 and 8 weeks of age. The mice were sacrificed at week 11 (Figure 4A), and the anti-HTT EM48 antibody was used to detect HTT aggregates in the brain and spinal cord tissues (Kakkar et al., 2016; Lee et al., 2017). Most of the HTT-positive aggregates/inclusions were present in the dorsal striatum (caudate nucleus, putamen) and the isocortex and to a lesser extent in other brain areas (e.g. hippocampal formation, brain stem olfactory areas, and cerebellum) and spinal cord. This distribution pattern is similar to previous reports (Dedeoglu et al., 2003; Ferrante, 2009). In the brain, the HTT aggregates were present mainly in the neuropil (neuropilar HTT aggregates) and to a lesser extent in the neurons (HTT intranuclear inclusions). Regarding the spinal cord, the amount of HTT protein aggregates was significantly lower than in the brain and was mostly present as intranuclear inclusions and rarely as neuropilar aggregates (Figure 4B).

Immunohistochemical analysis revealed that formation of HTT aggregates in the brain was reduced in mice treated with DNAJB6-enriched sEVs as compared with mice treated with control sEVs (i.e., XP-GFP sEVs). In the control brain tissues 0.5% of the total examined area was positive for inclusion bodies, whereas, in brain tissue of mice injected with XP-GFP-DNAJB6 sEVs, this area was reduced to 0.35% (Figure 4C), corresponding to a reduction of 31%. In spinal cord tissue, no significant difference between the extent of aggregation was found between XP-GFP-DNAJB6 sEV-treated and XP-GFP sEV-treated animals (Figure 4D). This might be explained by the already low aggregation levels in spinal cord in the XP-GFP sEV-treated animals (Figure S3), which may be due to the inherent low aggregation in spinal cord in R6/2 mice and/or efficacy of the endogenous chaperones in the XP-GFP sEVs.

Overall, the results show that DNAJB6-enriched C17.2 NSC-derived sEVs can suppress polyQ aggregation in the brain of R6/2 mice and encourage additional investigations on the use of sEVs as delivery vehicles to treat HD.

## Discussion

Prevention of the initiation of polyQ aggregation is an attractive strategy for therapies aimed at delaying the onset of HD. DNAJB6, unlike other chaperones, has been shown to bind to polyQ peptides or small oligomers and prevent their maturation into full fibrils as opposed to preformed polyQ aggregates, imparting functional benefits (Kakkar et al., 2016). Thus, DNAJB6 acts upstream of aggregate formation, which can potentially result in prevention or delay of pathological HTT aggregation and long-term protection against HD. Here, we used C17.2 NSC-derived sEVs for the delivery of DNAJB6 in cellular and mouse HD models. We show that such exogenous NSC sEV-based supplementation of the chaperone DNAJB6 can reduce the overall levels of polyQ aggregation *in vitro* and *in vivo*. The data reported are in line with studies in Drosophila showing that sEVs from cells overexpressing chaperones can be taken up by neighboring cells to contribute to protection against polyQ-caused neurodegeneration (Takeuchi et al., 2015), although other mechanisms may also contribute to such protection (Bason et al., 2019). Our data now extent these findings to exogenous delivery of cell-derived sEVs into mice and hereby provide proof-of-concept for the possibility that delivery strategies aimed at exogenously supplying molecular chaperones could hold promise as a therapeutic treatment for HD.

Because injection of purified chaperones directly into cerebrospinal fluid (CSF) would likely result in degradation of the chaperones by proteases/peptidases present in CSF, a delivery vehicle is required to protect the therapeutic cargo (DNAJB6) during its transport to its target cells. sEVs are attractive drug delivery vehicles for such purposes owing to their natural origin, low immunogenicity, ease of loading cargo by amongst others genetic engineering (Joshi et al., 2020), and target cell specificity (Ha et al., 2016; Hoshino et al., 2015; Thery et al., 2002; Tkach and Thery, 2016; Wiklander et al., 2015; Zomer et al., 2015). Moreover, sEVs have shown the capacity to penetrate from CSF into brain tissue (Ramirez et al., 2018). In addition, additional cargoes naturally present in sEVs might confer extra benefits, including endogenous chaperones (Takeuchi et al., 2015) or other cellular constituents such as e.g. GM1 and cholesterol, both enriched in sEVs (Skotland et al., 2017) and that have been shown to ameliorate HD symptoms *in vitro* and *in vivo* (Alpaugh et al., 2017; Valenza et al., 2015). This natural content of biological delivery vehicles, however, is difficult to control and may also introduce negative side effects.

The improved efficacy in reducing HTT aggregation of XP-GFP-DNAJB6 sEVs over XP-GFP sEVs, which already show anti-aggregation effects *in vitro*, supports an additional role for DNAJB6b over other endogenous content of EVs. However, in the present study in the *in vivo* experiments, a positive control group, i.e., R6/2 mice without treatment, and a vehicle control group, i.e., R6/2 mice intrathecally injected with PBS, were not included. As a consequence, a potential reducing effect of XP-GFP sEVs on polyQ aggregate formation cannot be excluded nor can we exclude a reducing effect of the injection procedure on polyQ aggregate formation. Definite proof that this effect is directly mediated by DNAJB6b delivered by sEVs requires additional studies. For example, comparing the effects of sEV-mediated delivery of a defective so-called M3-DNAJB6 mutant (Kakkar et al., 2016) may provide further insight. Inversely, one could test if XP-GFP sEVs derived from cells devoid of DNAJB6 would be less effective.

The effects observed both in our cellular and animal model are, albeit significant, modest in magnitude compared with e.g. what we found using intracellular overexpression of DNAJB6 (Kakkar et al., 2016), where about a 2- to 3-fold increase in total DNAJB6 levels was achieved. This could be likely explained by the yet too low dose of exogenous DNAJB6 delivered to the recipient cells. We cannot, at this stage, evaluate the sEV-mediated increase in DNAJB6 in the mouse experiments. However, looking at our cellular experiments, one must realize that HEK293 cells already show relatively high endogenous expression of DNAJB6. Incubation of cells with DNAJB6-enriched EVs increases the DNAJB6 content of the recipient cells by only ∼20% (quantified from blots in Figures 1C and 3C). The finding that this already has a detectable (additional) effect on reducing polyQ aggregation in wild-type recipient cells shows the potential of DNAJB6.

So, although this proof-of-concept study highlights the potential of sEVs as DNAJB6 carrier to decrease aggregation, multiple aspects need optimization and further research. Increasing the protein overexpression in the donor cells and further development of methods to reach higher protein loading efficiency of sEVs as well as employing cells that produce large(r) numbers of sEVs may contribute to get higher yields of EVs loaded with higher concentrations of DNAJB6. To obtain more effective delivery strategies further work will be required to increase the half-life of EVs in circulation, which is yet limited. As repeated injections may lead to immune activation, eventually compromising the health of the individual receiving treatment, studies are required to determine the optimal amount of EVs per dose and the dosing regimen (Wiklander et al., 2015).

Recently, hybrid liposome-sEVs have shown to be effective in reaching high levels of cargo delivery (Lee et al., 2016; Nakase and Futaki, 2015; Sato et al., 2016). Such designer hybrid sEVs may hold promise. But, given that even at these modest DNAJB6 levels aggregation is significantly reduced, it further not only highlights the effectiveness of this chaperone but also confirms how key the expression levels of DNAJB6 are for endogenous resistance to amyloid formation by polyQ proteins (Thiruvalluvan et al., 2020).

The anti-aggregation effects of DNAJB6 are not restricted to polyQ-huntingtin only. DNAJB6 works at the level of formation of polyQ oligomers independent of the context flanking the polyQ region (Hageman et al., 2010; Kuiper et al., 2017; Mansson et al., 2018), and thus its use can be extrapolated to other polyQ disorders such as spinocerebellar ataxia, spinal and bulbar muscular atrophy, and dentatorubropallidoluysian atrophy (Orr and Zoghbi, 2007). Recently, DNAJB6 was shown to also interact with the highly aggregation-prone amyloid-beta Aβ42 peptide (involved in Alzheimer disease) and efficiently inhibit amyloid aggregation (Mansson et al., 2014). Taken together, our proof-of-concept data show that sEV-mediated DNAJB6 delivery represents a potential strategy for the treatment of polyQ and other amyloid disorders, which warrants further investigation.

### Limitations of the study

In this article, the use of NSC-derived sEVs containing chaperone DNAJB6 as a potential therapeutic strategy for HD was evaluated. Using cellular HD models and the R6/2 mouse model, we show that DNAJB6-containing sEVs significantly reduce the extent of polyQ aggregation. However, there are several aspects that warrant further research in order to fully endorse the future use of chaperone-loaded sEVs for the treatment of HD. First, in our animal study, for the two treatment groups of mice all conditions were the same, except for the source of the sEVs, i.e., from GFP-expressing cells or from GFP-DNAJB6-expressing cells. This allowed us to ascribe differences between effects mediated by XP-GFP and XP-GFP-DNAJB6 sEVs to the elevated presence of DNAJB6 and/or related changes in EV content in the XP-GFP-DNAJB6 sEVs. However, we cannot and do not want to exclude that the sEVs from GFP-expressing cells may already have had some protective effects (as we see *in vitro*). Of note, NSCs express significant amounts of DNAJB6 (Thiruvalluvan et al., 2020), and thus EVs derived from GFP-only expressing NSCs may even be effective because of that, but also transfer of other protective factors cannot be excluded for such a protective effect either. The inclusion of a nontreated group of R6/2 mice would have been helpful in order to evaluate the potential therapeutic effect of XP-GFP sEVs. Likewise, a vehicle (PBS) control group is needed to assess possible effects of the intrathecal injection procedure.

Secondly, behavioral tests, e.g. rotarod, clasping, were not performed in this study, leaving the question if the observed antiaggregation effects are accompanied by a reduction in motor deficits unanswered. Investigation of the impact of XP-GFP and XP-GFP-DNAJB6 sEV treatment on the behavioral manifestations of the disease in R6/2 mice is required to assess the therapeutic potential of DNAJB6-loaded exosomes for HD treatment.

## STAR★Methods

### Key resources table

**Table:** 

REAGENT or RESOURCE	SOURCE	IDENTIFIER
**Antibodies**		

Rat monoclonal anti-Lamp2b ABL-93	Developmental Studies Hybridoma Bank	RRID: AB_2134767
Rabbit monoclonal anti-CD9	Abcam	ab92726; RRID: AB_10561589
Rabbir monoclonal anti-calnexin	StressGen	ADI-SPA-860; RRID: AB_10616095
Mouse monoclonal anti-GFP	Clontech	632381; RRID: AB_2313808
Rabbit polyclonal anti-mRFP/mCherry	Abcam	ab167453; RRID: AB_2571870
Rabbit polyclonal anti-DNAJB6	Custom made	NA
IRDye® 680RD Goat anti-Mouse IgG Secondary Antibody	Odyssey Li-COR	LI 926-68070; RRID: AB_10956588
IRDye® 800CW Goat anti-Rabbit IgG Secondary Antibody	Odyssey Li-COR	LI 926-32211; RRID: AB_621843
IRDye® 800CW Goat anti-Rat IgG Secondary Antibody	Odyssey Li-COR	LI 926-32219; RRID: AB_1850025
Goat anti-Rabbit IgG (H+L) Cross-Adsorbed Secondary Anitbody, Alexa Fluor 546	Invitrogen	A-11010; RRID: AB_2534077
Mouse monoclonal anti-EM48	Millipore	MAB5374; RRID: AB_10055116
Horse anti-mouse conjugated with biotin	Vector	PK-6100; RRID: AB_2336819

**Chemicals, peptides, and recombinant proteins**		

Doxycycline	Sigma	D9891

**Critical commercial assays**		

SG cell line 4D nucleofector kit	Lonza	V4XC-3032
Bio-rad DC protein assay kit	Bio-rad	5000114
ECL	GE-Healthcare	RPN2232

**Experimental models: Cell lines**		

Human HEK293T	Sigma Aldrich	12022001
Human HEK293T DNAJB6 K/O	Thiruvalluvan et al., 2020	N/A
Mouse C17.2 neural stem cells	Sigma Aldrich	07062902
Mouse C17.2-XPack-GFP	This paper	N/A
Mouse C17.2-XPack-GFP-DNAJB6b	This paper	N/A

**Experimental models: Organisms/strains**		

Mouse: R6/2: B6CBA-Tg(HDexon1)62Gpb/3J	The Jackson Laboratory	JAX: 006494
Mouse: CBA x C57Bl/6 F1 females (	Harlam Olac	B6CBAF1/OlaHsd

**Recombinant DNA**		

XPack CMV-XP-MCS-EF1-Puro	SBI biosciences	XPAK510PA-1
pcDNA5/FRT/TO GFP-DNAJB6b	Hageman et al., 2010	N/A
pCMV-XP-GFP-EF1-Puro	This paper	N/A
pCMV-XP-GFP-DNAJB6-EF1-Puro	This paper	N/A
pEGFP-Q74	Narain et al., 1999	Addgene plasmid #40262
pmRFP-Q74	This paper	N/A

**Software and algorithms**		

Nanoparticle Tracking Analysis (NTA) software 3.0	Malvern, Worcestershire, United Kingdom	N/A
Graph Pad Software Prism 5	GraphPad software, Inc.	https://www.graphpad.com/scientific-software/prism/

### Resource availability

#### Lead contact

Further information and requests for resources and reagents should be directed to and will be fulfilled by the lead contact, Inge Zuhorn (i.zuhorn@umcg.nl).

#### Materials availability

Newly generated materials are available upon request.

### Experimental model and subject details

#### Cell culture

C17.2 mouse neural progenitor cells were cultured in DMEM (Gibco, 41965-039) supplemented with 10% Fetal Bovine Serum (FBS, Bodinco, 5010), 5% Horse Serum (Invitrogen, 26050-088) and 1% Penicillin-Streptomycin sulfate (Gibco, 15140-122) at 37°C under 5 % CO_2_. Human embryonic kidney cells stably transfected with SV40 large T antigen (HEK293T) were cultured in DMEM (Gibco 41965-039) supplemented with 10% Fetal Bovine Serum (Bodinco, 5010) and 1% Penicillin-Streptomycin sulfate (Gibco, 15140-122) at 37°C under 5 % CO_2_.

#### *In vivo* animal model

All animal related procedures were performed in accordance with the University of Groningen Ethical Committee for Animal Experiments, which strictly adheres to the guidelines established by the European Convention for the Protection of Laboratory Animals. R6/2 transgenic mice were used for all the experimental procedures. Mice were housed in cages (3-4 mice/cage) under standard cage enrichment conditions and with *ad libitum* diet and water. Hemizygous R6/2 mice were bred in the University Medical Center animal facility by backcrossing R6/2 HD males to (CBA x C57Bl/6) F1 females (Harlan Olac, B6CBAF1/OlaHsd). For genotyping, ear cuts were taken from the pups, and genomic DNA was extracted using the prepGEM Tissue kit (Zygem, PTI0050). PCR was performed with primers R6/2 fwd/rev & HDAC4 fwd/rev. Primers Sequence (5’ – 3’): R6/2 fwd CGC AGG CTA GGG CTG TCA ATC ATG CT; R6/2 rev TCA TCA GCT TTT CCA GGG TCG CCA T; HDAC4 WT fwd CTT GTT GAG AAC AAA CTC CTG CAG CT; HDAC4 WT rev AGC CCT ACA CTA GTG TGT GTT ACA CA. During the entire study period, all animals were monitored for ill effects in terms of weight loss or deteriorated fur quality according to the Ethical Committee for Animal Experiments guidelines, and euthanized if excessive health deterioration was seen, which was not the case. The number of animals per treatment group was based on the resource equation method, using the formula E = total number of animals – number of groups, where 10 ≤ E ≤ 20. A minimum of 12 mice (6 per treatment group) was required. We used 8 animals per group. Males and females were equally divided over both treatment groups.

### Method details

#### Plasmids

XPack CMV-XP-MCS-EF1-Puro Cloning Lentivector was purchased from SBI biosciences (XPAK510PA-1). The XPack vector contains a peptide sequence that targets a protein to the interior exosomal membrane. GFP and GFP-DNAJB6 fragments were amplified from pcDNA5/FRT/TO GFP-DNAJB6b (Hageman et al., 2010). Each respective segment was inserted between XhoI and EcoRI to achieve pCMV-XP-GFP-EF1-Puro and pCMV-XP-GFP-DNAJB6-EF1-Puro respectively. pEGFP-Q74 was a gift from David Rubinsztein (Addgene plasmid #40262; http://n2t.net/addgene:40262; PRID:Addgene_40262). For mRFP-Q74 plasmid, EGFP was replaced for mRFP (Muller-Taubenberger et al., 2006) in the pEGFP-Q74 plasmid.

#### sEV donor cell lines

To generate the sEV donor cell lines XP-GFP and XP-GFP-DNAJB6, C17.2 cells were first transfected with pCMV-XP-GFP-EF1-Puro or pCMV-XP-GFP-DNAJB6-EF1-Puro by electroporation. Amaxa 4D nucleofection system (Lonza) was used for electroporation using SG transfection solution and program DN100 following the manufacturer's instructions. Transfected cells were then selected with Puromycin (1 μg/mL) until formation of single colonies. High XP-GFP or XP-GFP-DNAJB6 expressing single colonies were isolated and regrown under Puromycin (Sigma, P8833, 3 μg/mL) selection to create monoclonal stable cell lines. Alternatively, for the production of GFP-DNAJB6 containing sEVs, 4-5 × 10^6^ C17.2 cells were seeded in a 15-cm culture dish with complete medium and transfected the next day with pcDNA5/FRT/TO GFP-DNAJB6b plasmid using Lipofectamine 2000 (Invitrogen, 11668027) according to the manufacturer's protocol. For control sEVs, cells were transfected with pcDNA5/FRT/TO empty (control) plasmid. After 4 hours of incubation, cells were washed with HBSS (Gibco, 14025092) and serum free medium was replaced with EV depleted medium. Culture media were collected after 48 hours and sEVs were isolated as described above. For confocal microscopy imaging, 30 x 10^3^ HEK293T cells were seeded on glass cover slips (VWR, 631-1340) in a 48 wells plate coated with poly L-Lysine (Sigma, P-2636). For immunocytochemistry, 10 x 10^3^ wild type (WT), XP-GFP, and XP-GFP-DNAJB6b C17.2 cells were seeded on glass cover slips in a 48 well plate. For filter trap assays, 300 x 10^3^ HEK293T were seeded in a 6-wells plate (ThermoFisher Scientific) coated with poly L-Lysine. To generate DNAJB6 knockout (KO) HEK293T cells, cells were simultaneously transfected with DNAJB6 CRISPR/Cas9 KO(h) and HDR(h) plasmids using lipofection. Puromycin was introduced into the medium after 24 hours for antibiotic selection. Surviving cells were seeded as single-cells in a 96-well plate and expanded under antibiotic selection and screened for the absence of DNAJB6 (Thiruvalluvan et al., 2020). Cells were characterized for DNAJB6 by western blotting.

#### Preparation of EV-depleted medium

DMEM containing 10% FBS was centrifuged at 110,000 × g for 16 h at 4°C to deplete the fetal bovine serum-derived sEVs. The resulting supernatant was filter sterilized through a 0.2-μm filter (Millipore) and stored at 4°C.

#### sEV isolation and characterization

C17.2 sEV donor cells were seeded in T162 flasks (Corning). sEV depleted medium was added to the cells when they reached ∼40% confluence. 48 hours later, the medium was collected and sEVs were isolated by sequential centrifugation. Cells were removed from the supernatant by centrifugation at 500 g for 10 min, followed by centrifugation at 2,000 g for 10 min to remove cellular debris (Beckman Coulter, Allegra X-15R). Apoptotic vesicles and micro vesicles were removed by centrifugation at 10,000 g for 30 min in Sorvall Discovery 90SE ultracentrifuge using Beckman SW32i rotor. Finally, supernatant was subjected to ultracentrifugation at 110,000 g for 70 min to collect sEVs in the same rotor. sEV pellet obtained after this step was resuspended in 5 ml of PBS and pelleted again by ultracentrifugation at the same conditions using Beckman SW55i rotor. The final pellet was re-suspended in 50 μL PBS and protein concentration was measured with DC protein assay kit (Bio-Rad, 5000114). The size of particles was determined by recording and analyzing the Brownian motion of particles by NanoSight LM14 using Nanoparticle Tracking Analysis (NTA) software 3.0, Malvern, Worcestershire, United Kingdom following manufacturer’s protocol. For quality assessment, 20 μg whole cell lysates and sEV lysates were prepared by mixing the samples with Laemmli loading buffer with SDS and protease inhibitors (Roche, 11697498001). After boiling the samples at 90⁰C for 5 minutes, they were loaded in each well onto an SDS-PAGE gel, transferred to a PVDF membrane (Millipore, IPFL00010) and blocked with Odyssey blocking buffer (Li-COR, 927-40000) for 1 hour at RT. Blots were probed with primary antibodies in blocking buffer overnight at 4°C. Next, blots were washed with 0.1% PBS-Tween20 and then incubated in secondary antibody for 1 hour at RT. They were washed with 0.1% PBS-Tween20 and images were acquired with an Odyssey® Infrared Imaging System (Li-COR). Protein band intensities were measured using ImageJ.

#### Antibodies and reagents

For western blotting, primary antibodies against Lamp2b (rat; Developmental Studies Hybridoma Bank ABL-93; AB-2134767; 1:1000), CD9 (rabbit; Abcam ab92726; 1:1000), Calnexin (rabbit; StressGen ADI-SPA-860; 1:2000), GFP (mouse; Clontech 632381; 1:5000), mRFP/mCherry (rabbit; Abcam ab167453; 1:500) and DNAJB6 (rabbit; housemade; 1:2000) were used. Odyssey secondary anti-mouse, -rabbit and -rat antibodies (Li-COR, LI 926-68070, LI 926-32211 and LI 926-32219) were used at 1:5000 dilution. For immunocytochemistry, CD9 (1:100) was used followed by staining with secondary antibodies conjugated with Alexa 546 (goat; Invitrogen A-11010; 1:500). Antibodies for immunohistochemistry included EM48 (mouse; Millipore MAB5374; 1:200) and anti-mouse conjugated with biotin (horse; Vector BA9200; 1:300).

#### MTT assay

Viability of HEK293T after exposure to sEVs was evaluated by performing a 3-(4,5-dimethylthiazol-2-yl)-2,5-diphenyltetrazolium bromide (Sigma-Aldrich, M2128) assay. 10,000 HEK293T cells were seeded in 96-wells plates (ThermoFischer Scientific) precoated with PLL. Cells were treated with XP-GFP or XP-GFP-DNAJB6 sEVs in sEV depleted DMEM in triplicate (final volume of 200 μl), for 45 hours. Untreated cells incubated in sEV depleted DMEM were used as a negative control. During the final 3 hours of incubation, 20 μl MTT solution (5 mg/ml in 1X PBS) was added to cells. Thereafter, the medium was removed and formazan crystals were dissolved in 200 μl of DMSO (Sigma, 8418). The optical density of each well was measured after complete solubilization of the crystals, using a microplate spectrophotometer at 520 nm.

#### Immunocytochemistry

C17.2 cells were fixed 24 hours post seeding with 4% PFA in PBS for 30 min at RT and washed with PBS twice. Cells were permeabilized with 0.2% Tween-20 (Sigma, P1379) in PBS and rinsed with PBS before blocking with blocking buffer (3% BSA (Sigma, A7906)/PBS) for 1 hour at RT. Primary antibody against CD9 prepared in blocking buffer was added to cells and kept overnight at 4°C. The next day, cells were rinsed with PBS thrice and incubated with secondary antibodies and DAPI in blocking solution for 30min at RT. After rinsing with PBS, the coverslips were mounted on microscope slides using Faramount mounting medium and imaged with confocal microscopy (Leica SP8, HC PL APO CS2 63X, NA 1.4, oil immersion, filter settings for DiI; excitation 551nm, emission 565 nm and DAPI; excitation 358 nm, emission 463 nm, GFP; excitation 490nm, emission 544nm).

#### sEV uptake determination by fluorescence microscopy

To quantify sEV uptake, sEV membranes were labeled with the lipophilic dye DiI (Invitrogen, D282) by incubating purified sEVs with 1μM DiI solution in PBS for 5 min at RT. DiI labeled sEVs were collected by ultracentrifuging the reaction mixture at 100,000g for 70 min at 4°C and excess DiI was removed by washing with PBS. The supernatant was carefully aspirated and the pellet was resuspended in PBS. Then, the sEV protein content was measured using DC protein assay kit. HEK293T cells were incubated with or without DiI labeled 20 μg/ml sEVs in 200μl of medium for 36 hours, fixed with 4% PFA (in PBS, pH 7.4) and rinsed with PBS. For nuclear staining, DAPI (1μg/ml in PBS; Sigma, D9542) was added for 20 min at RT, and cells were rinsed with PBS and the coverslips were mounted using Faramount mounting medium (Dako, The Netherlands, S302580). Images were generated using confocal microscopy (Leica SP8, HC PL APO CS2 63X, NA 1.4, oil immersion) using filter settings for DiI (excitation wavelength as 551nm, emission wavelength as 565 nm) and DAPI (excitation wavelength as 358 nm, emission wavelength as 463 nm). For quantification, three random fields were chosen containing 6-10 cells for imaging and mean fluorescence intensity (MFI) of the DiI signal in the images was determined using ImageJ. The number of cells was counted for all images and the extent of uptake was calculated as MFI/number of cells.

#### Analysis of inclusion body formation of PolyQ proteins

HEK293T cells were transfected with pEGFP-Q74 (encoding EGFP-Htt(Q74)) using JetPEI® (Polyplus,10110-N) for 4h following the manufacturer’s protocol. Transfection efficiency was >70%. After rinsing the cells twice with HBSS, sEV depleted medium with or without 50 μg/ml sEVs in 1 ml of medium was added, and cells were incubated for 36 h. Cells were fixed with 4% PFA (in PBS, pH 7.4), rinsed with PBS and DAPI (1μg/ml in PBS; Sigma, D9542) was added for 20 min at RT directly followed by rinsing (PBS) before mounting the coverslips using Faramount mounting medium (Dako, The Netherlands, S302580). Samples were analyzed using fluorescence microscopy (Leica DMI 6000B, HCX PL FLUOTAR L, 40x, NA 0.60 dry, GFP excitation 490nm/emission 550nm, DAPI excitation 360nm/emission 460nm). The ratio of the number of cells with inclusion bodies to the total number of transfected cells presented the extent of inclusion body formation i.e. protein aggregation (n=3, >800 cells counted per experiment). Protein content of the donor cell line lysates and sEV lysates was determined with DC protein assay kit (Bio-Rad, 5000114). 20 μg of protein was heated for 5 min at 100°C after adding loading buffer and stored at -20°C until further use. SDS-PAGE was performed (10% gel, 100V, 2hr) and proteins were transferred to a polyvinylidene difluoride membrane (PVDF, Millipore, IPFL00010). The blots were blocked with Odyssey blocking buffer (Li-COR, 927-40000) for 1 hour at RT and followed by overnight incubation with primary antibodies prepared in blocking buffer at 4°C. The next day, blots were washed with 0.1% PBS-Tween20, incubated in secondary antibodies for 1 hour at RT and washed with 0.1% PBS-Tween20. Odyssey Infrared Imaging system (Li-COR) was used for imaging.

#### Analysis of HTT aggregation using Filter Trap Assay and Western blotting

HEK293T cell lysates were prepared as described above. For filter trap assay, after protein quantification (Bio-Rad, 5000114), serial dilutions were made to achieve final concentrations of 1μg/μl, 0.2 μg/μl and 0.01 μg/μl in filter trap assay buffer (10 mM Tris-HCl pH 8.0, 150 mM NaCl, 50 mM dithiothreitol and 2% SDS) and heated at 95°C for 10 min. 200μl of each dilution was then applied onto a cellulose acetate membrane. The membrane was washed with 0.1% PBS-Tween20 three times and blocked with 10% skimmed milk in 0.1% PBS-Tween-20 for 1 hour at RT, washed with 0.1% PBS-Tween20 thrice and stained with an anti-GFP antibody, followed by an HRP-conjugated anti-mouse secondary antibody. For western blotting, lysates of mRFP-Q74-expressing WT or DNABJ6 KO HEK293T cells treated with sEVs isolated from C17.2 cells transfected with empty or GFP-DNAJB6 plasmids. Transfection of WT or DNABJ6 KO HEK293T cells with the plasmid encoding mRFP-Htt(Q74) was performed using JetPEI® for 4h following the manufacturer’s protocol, before sEV addition. 30 μg (WT) and 20 μg (KO) protein was loaded per lane and SDS-PAGE was performed. After transfer, the membrane was blocked with odyssey blocking buffer for 1 hour at RT and incubated with primary antibody solution at 4°C overnight. The next day, the membrane was washed with 0.1% PBS-Tween20 thrice and stained with an appropriate secondary antibody.

#### Immunohistochemistry

Mouse brain and the vertebral column containing the spinal cord were fixed in 4% buffered formaldehyde. Vertebral columns were then decalcified (2 hours on a shaker, RDO RDO-04100, Klinipath) using a standard protocol. All tissues were then dehydrated, embedded in paraffin (2079A, Klinipath) and sliced into 4 μm sections using a microtome (HM340E, ThermoFischer). The sections were de-waxed and rehydrated in a series of decreasing alcohol concentrations. Antigen retrieval was performed by incubating the slides in 10mM citrate buffer (pH 6.0) for 15min. Endogenous peroxidase was blocked in 1% H_2_O_2_ in methanol for 30min. They were then labeled with anti-HTT antibody (EM48, 1:200, overnight at 4°C) followed by a secondary biotin carrying antibody (30min, RT). An avidin-biotin-complex (Vector; PK-6100; 30min; RT) and the chromogen (DAB, D5637, Sigma, 10min, RT) were added for visualization under bright field microscopy. Nuclear counterstaining was performed with hematoxylin for 30sec. Quantitative morphometric analysis was then performed on these sections. One slide per animal was scanned using a NanoZoomer slide scanner (Hamamatsu Photonics, Hamamatsu City, Japan). Whole scanned slide files were imported to the ImageJ program to measure the percentage (%) of positive EM48 staining. The percentage of positive staining was normalized automatically to the total examined surface area (mm^2^). Examined areas included all available tissue on the slides except for areas that showed artifacts (e.g. fragmented shredded areas).

#### Mice handling and intrathecal injections

Mice were treated with XP-GFP or XP-GFP-DNAJB6 sEVs in order to evaluate the effect of DNAJB6 on reduction of polyQ aggregation. For intrathecal injections, mice were anesthetized with isoflurane and positioned on a flat surface. A syringe with a 30-gauge needle (Hamilton, HAM7653-01) was used to inject the sEV suspension between the first and second lumbar vertebrae. The sEVs were slowly injected over a period of approximately 5 min and the needle was gently removed. 10 μl of a 5mg/ml sEV (XP-GFP-DNAJB6 or XP-GFP) formulation (total 50 μg protein) was used per injection. The injections were repeated twice after a 7-day interval each. After the injection, mice were returned to their cages and sacrificed 3 weeks after the 3^rd^ injection by lumbar dislocation. Brains and vertebral columns were harvested and immediately fixed in 4% paraformaldehyde for further processing. Four mice died during the intrathecal injection procedure, due to ill injection causing spinal column bleeding, and were excluded from the analysis. One spinal cord was damaged during sample processing, and therefore was not suitable for examination.

### Quantification and statistical analysis

Western blot experiments were performed twice and filter trap experiments were repeated 3 times. Statistical analysis was performed using ANOVA with Tukeys post hoc for cellular models and Wilcoxon test for R6/2 mice studies. Significant differences between groups are depicted as ∗p < 0.05, ∗∗p < 0.01, ∗∗∗p < 0.001 in the figure legends. GraphPad Prism version 5 was used for all statistical analyses.

## Data Availability

• All data reported in this paper will be shared by the lead contact upon request. • This paper does not report original code. • Any additional information required to reanalyze the data reported in this paper is available from the lead contact upon request.
