# Supplementary material for: DNAJB6b-enriched small extracellular vesicles decrease polyglutamine aggregation in in vitro and in vivo models of Huntington disease
Source: iScience. 2021 Oct 14;24(11):103282. doi: 10.1016/j.isci.2021.103282 (PMC8564107; doi:10.1016/j.isci.2021.103282)
Supplement: Document S1. Figures S1–S3 and Table S1 [file mmc1.pdf]

iScience, Volume *24*

Supplemental information

DNAJB6b-enriched small extracellular vesicles decrease polyglutamine aggregation in *in vitro* and *in vivo* models of Huntington disease

Bhagyashree S. Joshi, Sameh A. Youssef, Reinier Bron, Alain de Bruin, Harm H. Kampinga, and Inge S. Zuhorn

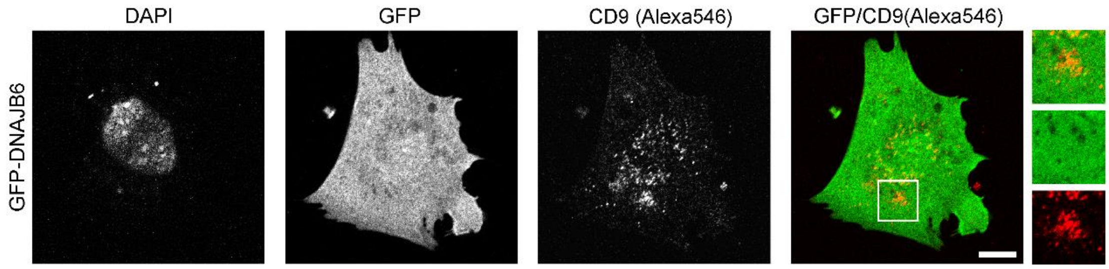

Figure S1 GFP-DNAJB6 lacking XPack tag remains cytosolic, Related to Figure 1. Cells expressing GFP-DNAJB6b without the XPack tag. Note that GFP-DNAJB6b is found in the nucleus and cytosol in line with DNAJB6b endogenous localization and shows minimal colocalization with CD9. Bar: 10 µm.

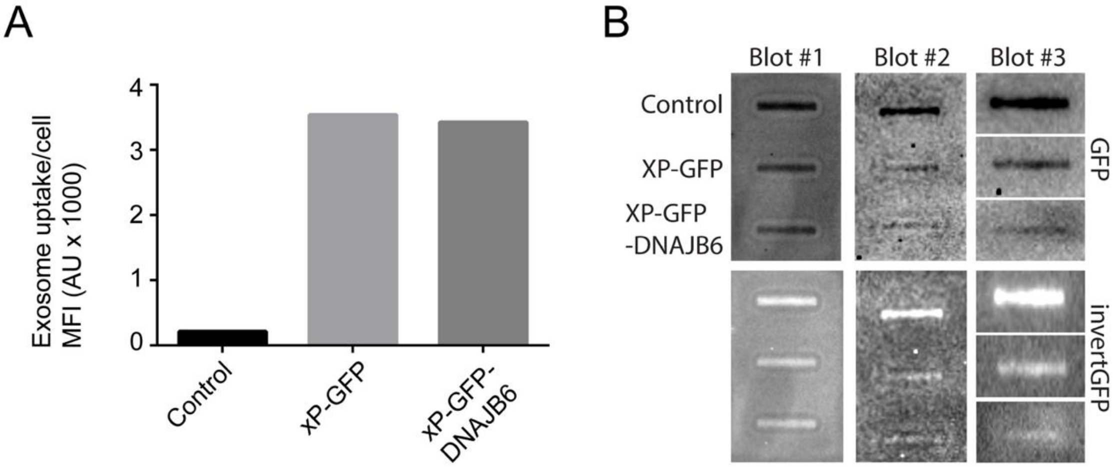

Figure S2 XP-GFP and XP-GFP-DNAJB6 sEVs are internalized by recipient cells to a similar extent and efficiently reduce polyQ aggregation *in vitro*, Related to Figure 3. (A) EGFP-Q74 HEK293T cells were incubated with DiI-labeled XP-GFP or XP-GFP-DNAJB sEVs for 48 hr and investigated by fluorescence microscopy. Images were captured from three randomly selected fields of view, each containing 6-10 cells. Mean fluorescence intensity of DiI per cell was determined using ImageJ. Representative images are shown in Figure 2b**.** (B) Three filter trap blots used for quantification of insoluble aggregates in Figure 3 (I). For blot #1 intensity values were corrected to remove the influence of the lighter shade at the bottom left of the blot. *XP: XPack; AU: arbitrary units, MFI: mean fluorescence intensity.*

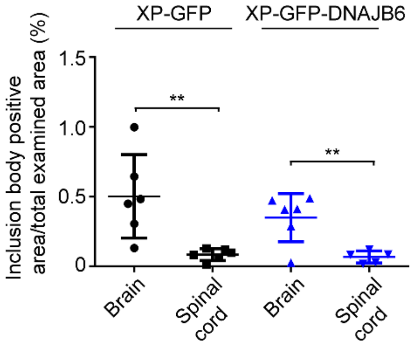

Figure S3 R6/2 mice treated with XP-GFP and XP-GFP-DNAJB6 sEVs show less HTT aggregation in spinal cord tissue than in brain, Related to Figure 4*.* Mutant HTT aggregation is significantly lower in spinal cord tissues in comparison with the brain tissues in all animals. *XP:* XPack.

Table S1 Increase in DNAJB6b in sEVs upon DNAJB6b overexpression in producer cells, Related to Figures 1 and 2. Ratio of DNAJB6b levels in sEVs from DNAJB6b-overexpressing cells and control cells, determined by intensity of DNAJB6b protein bands (50 kDa *vs* 25 kDa) on Western blot.

SEV samples	Ratio of DNAJB6b band intensities
**Figure 1B**	
GFP-DNAJB6/Mock	5.27
**Figure 2D**	
XP-GFP/WT	1.09
XP-GFP-DNAJB6/WT	12.79
XP-GFP-DNAJB6/XP-GFP	11.70
